# ChokeSafe: Empowering Children with Life-Saving Choking-Management Skills

**DOI:** 10.3390/children11030299

**Published:** 2024-03-02

**Authors:** Eleana Tse, Katerina Plakitsi, Spyridon Voulgaris, George A. Alexiou

**Affiliations:** 1 Department of Neurosurgery, School of Medicine, University of Ioannina, 45500 Ioannina, Greece; e.tse@uoi.gr (E.T.); svoulgar@uoi.gr (S.V.); 2 Department of Early Childhood Education, School of Education, University of Ioannina, 45110 Ioannina, Greece; kplakits@uoi.gr

**Keywords:** children, choking management, schoolteachers, educational program

## Abstract

Choking stands as the fourth leading cause of unintentional injury deaths. This research aimed to evaluate the ability of young schoolchildren to grasp and remember choking-management techniques, as well as to compare the effectiveness of instructors. We conducted a randomized controlled trial to assess the impact of choking training on young children. We randomly selected 180 children aged 4–8 years and divided them into a training group (120 children) and a control group (60 children). We evaluated the students’ response to a choking incident with a specific scenario one day before, one day after, and two and seven months after the training, as well as once to the control group. Before the training, there was no significant difference between the groups. However, after the training, the training group’s scores showed a significant increase compared to their pre-training scores and those of the control group. Even at two- and seven-month post-training, the training group’s scores had decreased but remained higher than their pre-training scores and those of the control group. Choking training can benefit young children. Our research highlights the equal importance of both regular classroom teachers and specialized personnel in imparting these essential skills. However, further research is necessary to confirm these observations and explore methods for sustaining the acquired knowledge from the training.

## 1. Introduction

Each year, a significant number of lives are lost globally because of choking incidents, underscoring the urgent need for our focused consideration [1,2,3,4]. Choking stands as the fourth leading cause of unintentional injury deaths, underscoring the urgent need for heightened awareness and education to prevent these devastating fatalities [1]. In the United States, 5051 people lost their lives due to choking-related incidents in 2015, showing how widespread and severe the problem is [1]. In the UK, there was a worrying 17% increase in choking-related deaths in 2016 compared to the previous year, indicating a need for better prevention and response strategies [2]. A study found that 4.7% of children die annually due to choking incidents in Thailand, emphasizing the vulnerability of young people to this danger [3]. These numbers underscore the importance of raising awareness, improving safety measures, and ensuring proper emergency response to prevent choking incidents and save lives.

Educational interventions aiming at preventing choking incidents play a pivotal role in filling knowledge gaps and have proven their efficacy in reducing injury rates [4,5,6,7]. Research conducted by Bentivegna and colleagues [6] involving 202 participants showcased a significant improvement in knowledge regarding choking hazards and prevention immediately after the participants viewed an educational video—an enhancement that endured even 30 days later. Similarly, Elfeshawy and colleagues’ study demonstrated that a substantial majority of mothers maintained a satisfactory level of knowledge even a month after participating in an educational program [4].

While numerous educational interventions have been developed for cardiopulmonary resuscitation [8,9,10,11,12,13,14,15,16,17], studies specifically addressing choking management in younger children remain limited. In a study by Wafik and Tork, nursing students taught first aid skills to preparatory school children, focusing on how to respond when someone is choking [7]. The outcomes revealed a substantial enhancement in the children’s proficiency in managing choking emergencies. This underscores the pivotal role of structured educational interventions in empowering children with the necessary skills and confidence to respond effectively in critical situations. Such findings underscore the potential of educational initiatives to equip children with life-saving capabilities, thereby bolstering their readiness to contribute to public health and safety endeavors.

Additionally, the research by Riesmiyatiningdyah et al. [14] focused on sixth graders and discovered a big boost in their understanding of first aid, especially when dealing with choking emergencies. This emphasizes the need for teaching first aid methods that match a child’s age. It demonstrates how teaching kids the appropriate skills at the right stage of development can give them crucial knowledge and abilities that might help save lives. Such studies highlight the importance of tailoring educational efforts to the specific needs and capacities of young learners, ultimately contributing to their preparedness and safety in real-life situations.

Furthermore, the study conducted by Wilks and Pendergast examined the efficacy of teaching 11 to 12-year-old children how to respond when someone is choking [15]. The results were promising, indicating a substantial improvement in the children’s abilities after the training. This research highlights the transformative potential of teaching programs, illuminating the significant positive impact they can have on children’s preparedness, making them not only safer but also more confident when faced with choking emergencies.

This study was specifically designed to assess the efficacy of an educational program tailored for children aged 4 to 8 enrolled in Greek schools. The research sought to address the following questions:

1. Can primary school-aged children, ranging from 4 to 8 years, effectively acquire the essential skills required to manage choking incidents?
2. What is the duration over which these young children can retain their knowledge and skills concerning choking incident management?
3. To what degree does the efficacy of instruction differ between classroom teachers and specialized personnel when imparting knowledge and skills related to choking management to young children?

By addressing these research questions, the study aimed to offer valuable insights into the learning and retention abilities of young schoolchildren regarding choking management techniques, as well as to compare the effectiveness of various instructors in delivering this crucial information.

## 2. Materials and Methods

We conducted an experimental study with an educational program in randomly selected schools in Thesprotia, Greece. Children aged 4–8 were asked to join after obtaining written permission from their parents. We randomly selected 180 children. For the calculation of the sample, the Gpower 3.1.9.7 software was utilized. With a power of 80%, 159 students from Primary Education attending public elementary schools (for children aged 6–12 years) and kindergartens (for children aged 4–5 years) in the Regional Unit of Thesprotia were initially selected to form the study sample. However, we increased the sample size to account for possible losses that may occur during the research. Therefore, 180 students participated, with each group comprising 60 students. The study involved the participation of six schools—three (3) primary schools and three (3) kindergartens. Participants were randomly assigned to three groups: a control group and two experimental groups, one trained by teachers and the other by first aid experts. The experimental groups divided into 12 subgroups with approximately 10 students per instructor. The control group received no intervention, serving as a reference to ensure that all groups had the same level of knowledge and skills.

Scenario-based observation has been chosen as a reliable and proven method for assessing students’ first aid skills. This approach provides a practical and realistic experience for students, allowing them to face real-life situations they may encounter. Through this observational method, we can evaluate students’ performance, their understanding of fundamental principles and correct first aid procedures, and the impact of practical training on their skill development. The specific scenario we have chosen for assessment involves evaluating the students’ response to a choking incident. In this scenario, there is a girl named Eleni who is choking while eating an apple. Initially, she begins to cough, but then stops coughing, clutching her throat and unable to speak. Eleni was a child participant from every school, but she did not take part in the training sessions. Specifically, at the kindergarten level, Eleni was 5 years old, while at the primary school level, she was between 6 and 7 years old. The scenario was the same at all time points. First aid skills were assessed by the researcher using a checklist. The checklist consisted of 15 items. Two covered demographics, and the remaining items were designed for the assessment of choking management and calling an ambulance (see Appendix A). More specifically, the assessment of the correct positioning of the hands and the execution of abdominal trusts was part of the evaluation process used in our study. A score of one (1) was assigned when the skill was demonstrated, whereas a score of zero (0) was given when the skill was not demonstrated.

In accordance with the confidentiality regulations of the Greek national education system, the researchers did not have access to the personal data of the children.

The main research commenced in early November 2022 and wrapped up in early June 2023. The training spanned 6 instructional hours, with sessions conducted at a rate of 2 instructional hours per week. The experimental groups were assessed four times: before the intervention, immediately after its completion, two months later, and seven months after the intervention. On the other hand, the control group was evaluated only once, before the intervention. The study has been registered as a clinical trial under the identifier NCT05563129 as of 3 October 2022.

### 2.1. Training Program

The training program for choking was designed to be a single 45 min session, which included both theoretical instruction and hands-on practical exercises. The theoretical components were delivered through engaging electronic presentations, ensuring comprehensive coverage of essential concepts. In parallel, the practical segment of the session involved roleplay exercises. Participants actively engaged in simulated scenarios, allowing them to apply the theoretical knowledge in a realistic and interactive setting. Additionally, teachers received a comprehensive 2 h first aid training session. The program was specifically customized to suit the age group of the children involved in the study. The training program was the same for all groups.

### 2.2. Ethical Considerations

The study received approval from the University of Ioannina Ethics Committee. Prior to commencing the study, all participating children, their parents, and the involved teachers were provided with both written and verbal information about the study. Parents were informed of their right to withdraw their child at any time, ensuring complete anonymity. Written informed consent was obtained from parents for their child’s participation in the study.

### 2.3. Data Analysis

The analysis was conducted utilizing SPSS software version 28.0, with a significance level established at 0.05 for all cases. Data from the study are presented as percentages, accompanied by 95% confidence intervals. Descriptive statistics comprise percentages and means. Pairwise comparisons were employed to assess variations between different measurement instances for each training group. Normality assessments were executed utilizing the Shapiro–Wilk criterion and relevant QQ plots.

## 3. Results

The study enrolled 180 students, with an average age of 6 years of age. In each group, boys constituted around 48–49%, while girls comprised approximately 51–52% of the participants. In the “Training by Teachers” group, children ranged from 4 to 8 years old, averaging about 6.02 years, with some variation around 1.07 years. Similarly, in the “Training by Specialized Personnel” group, children were also aged 4 to 8, averaging around 6.00 years, with a bit less variation around 0.99 years. Lastly, the “Control Group” had children aged 4 to 8, averaging around 5.90 years, with a similar variation of about 0.99 years. Each group in the pretest participants did not know how to manage choking. This indicates that participants had limited knowledge or were unaware of choking management before the training. After the training, the participants’ reactions significantly improved in the post-test. Except for 4, 8, and 13, all participants correctly applied the choking process (Table 1). After 2 and 7 months, performances decreased compared to the post-test, but still, the training produced positive results.

**Figure 1:**
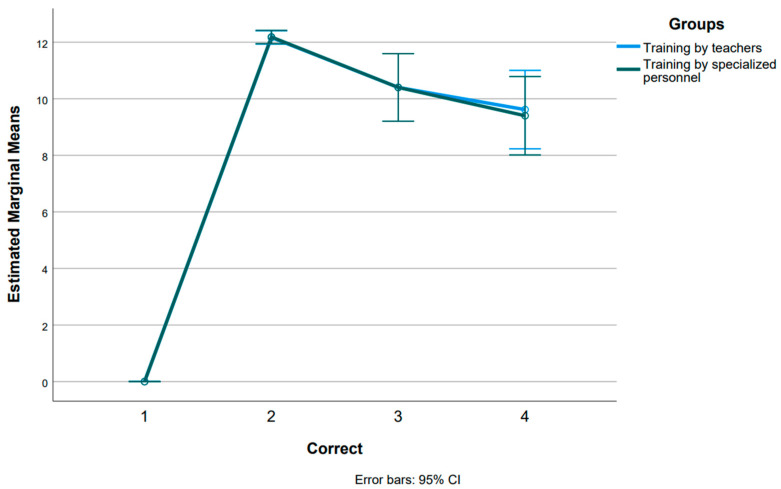
Differences between the various measurement points for each training group.

In the comparison of pretest results among the three groups, no statistically significant difference was observed, indicating that all participants initially exhibited a similar lack of proficiency in choke management. Following the training intervention, both training groups demonstrated noticeable improvements in knowledge. Furthermore, the statistical analysis revealed no significant difference (*p* = 0.921) between the two training groups post-intervention. In comparing the results of the two training groups at the measurement point after 2 months (Follow-up at 2 months), no statistically significant differences are observed (*p* = 1.000). In comparing the results of the two groups at the measurement point after 7 months (Follow-up at 7 months), again, there is no statistically significant difference (*p* = 0.827). Overall, these results indicate that there are no statistically significant differences in performance between the two training groups, nor at different measurement time points. Participants in both groups significantly improved their performance during the training and maintained this improvement in subsequent follow-ups (Figure 1).

Table 2 presents the results of comparisons among various measurement points (Pretest, Post-test, Follow-up at 2 months, and Follow-up at 7 months) for each training group (“Training by Teachers” and “Training by Specialized Personnel”). For the “Training by Teachers” group, all comparisons are statistically significant (*p* < 0.050), except for the comparison between the results of “Follow-up at 2 months” and “Follow-up at 7 months”. This means that the difference between these two measurement points, “Follow-up at 2 months” and “Follow-up at 7 months”, is not statistically significant. Participants’ performances at these two time points are similar, and there is no significant difference between them at the 95% confidence level. For the “Training by Specialized Personnel” group, all comparisons are statistically significant (*p* < 0.050), except for the comparison between the results of “Follow-up at 2 months” and “Follow-up at 7 months”. This means that the difference between these two measurement points, “Follow-up at 2 months” and “Follow-up at 7 months”, is not statistically significant.

Table 3 illustrates the performance differences among age groups at various time points, focusing on children aged 4–5 years old compared to those aged 6–8 years old. Throughout all time points, children aged 6–8 consistently demonstrate higher mean values than their younger counterparts. During the pretest phase, the mean difference between the 4–5 age group and the 6–8 age group was statistically significant (*p* < 0.001). This trend persisted in subsequent assessments, including the post-test, where the mean difference remained significant (*p* < 0.001). Similarly, in follow-up assessments at 2 months and 7 months, children aged 6–8 continued to exhibit higher mean values compared to those aged 4–5, with statistically significant differences observed (*p* < 0.001). Overall, these results highlight that those older children, aged 6–8, consistently performed better than younger children, aged 4–5, across different times.

## 4. Discussion

This study represents a significant contribution to the field of first aid, particularly concerning children aged 4 to 8 years old. By focusing on this specific age group, our research aims to address a crucial gap in the literature regarding appropriate first aid interventions tailored to the developmental needs and capabilities of young children.

The results showed that children aged 6–8 years performed better than those aged 4–5 years across all time points. This suggests that age influences performance levels. Older children likely have had more time to develop and refine the skills being assessed, leading to higher scores. The findings from the study conducted by Lubrano et al. [17] revealed a significant difference in performance among children across different school classes. Specifically, the evaluation outcomes indicated that older children, notably those in the V school class, achieved superior scores compared to their counterparts in the IV and III classes, with statistical significance observed (*p* < 0.001). This suggests an association between age or grade level and academic performance, with older students demonstrating higher levels of achievement in the assessed skills.

The study conducted by Alhidayat and Latif [16] yielded comparable outcomes. They investigated the effectiveness of utilizing both demonstration and roleplay techniques to augment understanding of choking management among junior high school students. Employing purposive sampling, the study encompassed a group of 60 junior high school students. Results indicated a noteworthy enhancement in choking-management skills among the students through the combined use of demonstration and roleplay methods.

While numerous studies have concentrated on instructing children in cardiopulmonary resuscitation (CPR), basic life support, and emergency response protocols, this emphasis aligns with prior research by Reveruzzi et al. [18], underscoring the prevalence of CPR and basic life support training. While these skills are undoubtedly vital, it is equally crucial to encompass other essential areas of knowledge, such as choking prevention.

The results from our repeated measurements revealed that some trained students had forgotten specific information. However, what remains significant is that despite the increase in incorrect answers compared to the initial phase of training, their response levels remained higher than both the initial measurement (pretest) and those of untrained students in the control group. This observation reaffirms the substantial improvement in knowledge and skills among the trained students throughout the educational program.

Educating children in first aid is crucial, and educators play a key role in this process [8]. The focus has primarily been on specialized personnel. However, our research underscores that both classroom teachers and specialized personnel equally contribute to imparting these crucial skills. Classroom teachers have demonstrated their ability to effectively convey first aid knowledge and skills to their students [19]. Their adeptness at simplifying complex procedures into understandable explanations makes the educational experience engaging and constructive for students. This revelation leads us to the conclusion that classroom teachers can significantly contribute to teaching first aid in schools. With adequate training and support, they can foster safe environments within schools and prepare students for emergency situations. This not only reduces training costs but also expands the number of individuals receiving this vital education.

It is crucial to emphasize that when teaching first aid to children, it is essential to consider their age-specific characteristics [19,20]. Research underscores the significance of tailoring teaching methodologies to suit the developmental stages of young learners [19,20]. For younger children, interactive games and activities serve as invaluable tools for imparting essential safety and first aid knowledge while keeping them engaged and receptive [16]. Interactive approaches, such as roleplaying games, hold merit in capturing children’s interest and facilitating experiential learning. Through these simulated emergency scenarios, children not only absorb vital first aid skills but also cultivate confidence in their ability to respond effectively in real-life situations. By integrating these educational activities seamlessly into play, educators can foster a positive association with first aid concepts, instilling a sense of responsibility and readiness in children to address emergencies proactively. Ultimately, by recognizing and harnessing age-specific learning preferences, educators can empower children with the knowledge and skills necessary to navigate potential emergencies with competence and assurance.

Integrating first aid education into the curriculum of children can serve as a cornerstone of comprehensive educational programs. By embedding first aid training into existing curricula, we not only democratize access to this critical knowledge, regardless of socio-economic background, but also underscore the importance of these life-saving skills within the educational paradigm. We advocate for the compulsory inclusion of first aid programs in school curricula, ensuring that every student receives instruction in these essential skills. By mandating first aid education in schools, we affirm the value of preparedness and response capabilities as integral aspects of a well-rounded education. Such a proactive approach not only equips students with the practical skills needed to address emergencies but also fosters a culture of safety, empathy, and community awareness. Moreover, by instilling these skills at a young age, we lay the foundation for a generation empowered to act decisively in times of crisis, potentially saving lives and promoting public health and safety.

The integration of first aid education into the educational framework yields multifaceted benefits, extending beyond individual students to encompass broader societal well-being. This proactive initiative prioritizes the cultivation of a generation equipped with both the theoretical understanding and practical skills necessary to adeptly manage emergency situations. By instilling comprehensive first aid knowledge and fostering confidence in response capabilities among students, we lay the groundwork for a safer and more resilient society. Through this strategic approach, we address not only the immediate needs of individuals in emergency scenarios but also contribute to the overall public health infrastructure. By empowering individuals with the ability to effectively respond to emergencies, we mitigate the severity of injuries, reduce the burden on healthcare systems, and potentially save lives. Furthermore, by promoting a culture of preparedness and proactive intervention, we cultivate a societal ethos that values collective well-being and community resilience.

Schools represent cost-effective educational institutions accessible to a wide demographic, facilitating the acquisition of knowledge across diverse populations [21,22,23]. Both students and teachers function as “multipliers” within first aid training programs, extending the impact of individuals trained in first aid [22]. For instance, to significantly reduce fatalities attributed to cardiac arrest, it is projected that a minimum of 15% of the population should receive CPR training [20]. However, attaining this objective solely through voluntary courses organized by trainees or trainers presents notable challenges [24]. Therefore, schools represent an optimal setting for implementing first aid training due to their structured environment and widespread accessibility, facilitating the dissemination of vital life-saving knowledge and skills.

## 5. Limitations

It is essential to address the limitations of the study to ensure a proper interpretation of the findings and to provide recommendations for future research to overcome these limitations and further explore the topic. One limitation of the study could be the lack of long-term follow-up to assess the retention of choking prevention knowledge and skills among the trained students. Long-term follow-up assessments would provide valuable insights into the effectiveness of the educational program over an extended period, allowing for a more comprehensive evaluation of its impact. Another limitation is that the control group was assessed only once. Finally, our study lacked blinding in the assessment process. This means that observers knew when assessments were conducted, which could introduce bias into the results.

## 6. Conclusions

Our study emphasizes the significance of acquiring first aid skills across all age groups, underscoring the necessity for widespread first aid training. Our research specifically focused on the impact of choking training in young children through a randomized controlled trial. There was no notable difference between the three groups, but following the training, the training groups exhibited a significant improvement in their response scores, surpassing both their pre-training levels and those of the control group. Even at the two- and seven-month follow-ups, the training group’s scores, while reduced, remained higher than both their initial scores and those of the control group. Our study highlights the equal contribution of regular classroom teachers and specialized personnel in imparting these crucial skills. These findings provide convincing evidence for the effectiveness of choking training among young children. Commencing the course at age 5 and conducting annual revisions could be a promising strategy. Nonetheless, further research is essential to validate these results and explore methodologies for maintaining the retention of acquired knowledge over extended periods.

## Figures and Tables

**Table 1:** Post-test results for each training group.

	Groups							
	Training by Teachers				Training by Specialized Personnel			
	Wrong		Correct		Wrong		Correct	
	N	%	N	%	N	%	N	%
1	0	0.0%	60	100.0%	0	0.0%	60	100.0%
2	0	0.0%	60	100.0%	0	0.0%	60	100.0%
3	0	0.0%	60	100.0%	0	0.0%	60	100.0%
4	16	26.7%	44	73.3%	17	28.3%	43	71.7%
5	0	0.0%	60	100.0%	0	0.0%	60	100.0%
6	0	0.0%	60	100.0%	0	0.0%	60	100.0%
7	0	0.0%	60	100.0%	0	0.0%	60	100.0%
8	22	36.7%	38	63.3%	17	28.3%	43	71.7%
9	0	0.0%	60	100.0%	0	0.0%	60	100.0%
10	0	0.0%	60	100.0%	0	0.0%	60	100.0%
11	0	0.0%	60	100.0%	0	0.0%	60	100.0%
12	0	0.0%	60	100.0%	0	0.0%	60	100.0%
13	12	20.0%	48	80.0%	15	25.0%	45	75.0%

**Table 2:** Differences between the various measurement points for each training group.

Groups	(I) Correct	(J) Correct	*p*
Training by teachers	Pretest	**Post-test**	<0.001
		Follow-up at 2 months	<0.001
		Follow-up at 7 months	<0.001
	Post-test	Follow-up at 2 months	0.020
		Follow-up at 7 months	0.001
	Follow-up at 2 months	Follow-up at 7 months	0.317
Training by specialized personnel	Pretest	Post-test	<0.001
		Follow-up at 2 months	<0.001
		Follow-up at 7 months	<0.001
	Post-test	Follow-up at 2 months	0.018
		Follow-up at 7 months	<0.001
	Follow-up at 2 months	Follow-up at 7 months	0.084

**Table 3:** The performance differences among age groups at different time points.

	Age Group		Mean Difference	*p*
Pretest	4–5	6–8	0.000	
	6–8	4–5	0.000	
Post-test	4–5	6–8	−0.862	<0.001
	6–8	4–5	0.862	<0.001
Follow-up at 2 months	4–5	6–8	−3.225	<0.001
	6–8	4–5	3.225	<0.001
Follow-up at 7 months	4–5	6–8	−5.900	<0.001
	6–8	4–5	5.900	<0.001

## Data Availability

Data are contained within the article.

## Appendix A

	**Checklist**
**Gender:**	
**Age:**	
**Encouraging Coughing**	
1. Encourages the person to cough.	
**Back Blows**	
2. Begins back blows after the person stops coughing.	
3. Administers 5 back blows.	
4. Administers back blows correctly.	
5. Checks after each blow if the foreign object came out.	
**Abdominal Thrusts**	
6. Continues with abdominal thrusts after back blows.	
7. Administers 5 abdominal thrusts.	
8. Administers abdominal thrusts correctly.	
9. Checks after each thrust if the foreign object came out.	
**Patient Collapses and is unresponsive**	
10. Calls for an ambulance immediately.	
11. States their name.	
12. Describes the incident.	
13. Provides the address.	
